# A general representation scheme for crystalline solids based on Voronoi-tessellation real feature values and atomic property data

**DOI:** 10.1080/14686996.2018.1439253

**Published:** 2018-03-19

**Authors:** Randy Jalem, Masanobu Nakayama, Yusuke Noda, Tam Le, Ichiro Takeuchi, Yoshitaka Tateyama, Hisatsugu Yamazaki

**Affiliations:** a Japan Science and Technology Agency (JST), PRESTO, Saitama, Japan; b National Institute for Materials Science – Global Research Center for Environment and Energy Based on Nanomaterials Science (NIMS – GREEN), Tsukuba, Japan; c Center for Materials Research by Information Integration (CMI^2^), Research and Services Division of Materials Data and Integrated System (MaDIS), National Institute for Materials Science – ‘Materials Research by Information Integration’ Initiative (NIMS – Mi^2^i), Tsukuba, Japan; d Frontier Research Institute for Materials Science, Nagoya Institute of Technology, Nagoya, Japan; e Elements Strategy Initiative for Catalysts and Batteries, Kyoto University, Kyoto, Japan; f Department of Computer Science, Nagoya Institute of Technology, Nagoya, Japan; g RIKEN Center for Advanced Intelligence Project, Tokyo, Japan; h Battery Material Engineering & Research Division, Toyota Motor Corporation, Susono, Japan

**Keywords:** Materials informatics, material descriptors, machine learning, inorganic solids, crystalline solids, density functional theory, 60 New topics/Others, 401 1st principle calculations, 404 Materials informatics / Genomics

## Abstract

Increasing attention has been paid to materials informatics approaches that promise efficient and fast discovery and optimization of functional inorganic materials. Technical breakthrough is urgently requested to advance this field and efforts have been made in the development of materials descriptors to encode or represent characteristics of crystalline solids, such as chemical composition, crystal structure, electronic structure, etc. We propose a general representation scheme for crystalline solids that lifts restrictions on atom ordering, cell periodicity, and system cell size based on structural descriptors of directly binned Voronoi-tessellation real feature values and atomic/chemical descriptors based on the electronegativity of elements in the crystal. Comparison was made vs. radial distribution function (RDF) feature vector, in terms of predictive accuracy on density functional theory (DFT) material properties: cohesive energy (CE), density (*d*), electronic band gap (BG), and decomposition energy (Ed). It was confirmed that the proposed feature vector from Voronoi real value binning generally outperforms the RDF-based one for the prediction of aforementioned properties. Together with electronegativity-based features, Voronoi-tessellation features from a given crystal structure that are derived from second-nearest neighbor information contribute significantly towards prediction.

## Graphical abstract

**Figure:**
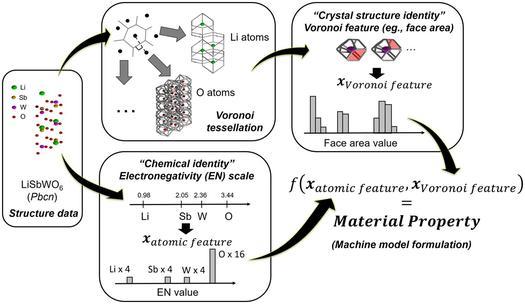

## 1. Introduction

Materials informatics in tandem with high-throughput density functional theory (DFT) calculations has now become increasingly deployed for exploration, optimization, and property forecasting of inorganic materials. This became evident with the emergence of DFT databases containing a large number of entries on physical and thermodynamical properties, for example, for data mining and material screening tasks [1–3]. Aside from the wealth of material data-sets, the inorganic materials field has also greatly benefited from advances made in machine learning algorithms and computer hardware performance. Practical demonstrations and successes have since been shown in areas such as Li-ion batteries [4–9], photovoltaic devices [10], catalysis [11,12], thermoelectrics [13,14], solar cells [15], superconductors [16], and data storage [17].

The underlying assumption in the use of informatics tools that affords learning of structure–property relationships is the effectiveness and validity of the input data representation for materials, that is, descriptors [18]. The choice and formulation for this is often far from being trivial but in common practice, it is usually guided by prior knowledge of a given application domain or by chemical intuition. For example, we used local structure descriptors and atomic/chemical descriptors in our previous works in order to efficiently predict a transition state property called Li ion migration energy which is a useful metric for assessing fast ionic conduction in candidate compounds for use in battery applications [8,18–20]. Although the structure descriptors were proven predictive for the aforementioned target property, most of them were inherently constrained and usable only to a few structure types at a time. For more general chemical search spaces or machine learning tasks though, structure descriptors need to be unrestricted with respect to structure type. Meanwhile, it is worth mentioning that atomic or chemical descriptors have been used as well by others with notable success for a variety of materials property predictions [18,21,22].

In the bio- and chem-informatics field, chemical graph theoretical matrices are commonly applied to represent molecules in a general manner [23]. Not surprisingly, some of the early representations for inorganic materials were inspired from efforts such as this, example is the so-called Coulomb matrix (CM) which contains information on nuclear charge *Z* and atom *I*-atom *J* pair distance *r* [24,25]:

(1) $$m_{I-J}=\left\{\begin{array}{ccc} 0.5Z_{I-J}^{2.4} & \text{for} & I=J\\ \frac{Z_{I}Z_{J}}{\left|r_{I}-r_{J}\right|} & \text{for} & I\neq J \end{array}\right.,$$

where *m*_*I*–*J*_ denotes matrix element. Essentially, the method is a form of descriptor *binning* with the matrix elements *m*_*I*–*J*_ assigned for atom-atom pair bins. However, the direct use of input representations from organic molecules for inorganic materials is usually inappropriate and ineffective because of 3D periodicity issue and dependency on the number of atoms and atom ordering. CM was combined with Bravais matrix (i.e. information from primitive translation vectors and unit cell basis) as a feature vector but the predictive accuracy is unsatisfactory, e.g. for the value of the density of states at the Fermi energy (DOS_F_) [26].

Alternative representations for inorganic crystals have since been proposed, again, within the concept of binning material features. One example is the concatenation of a series of radial distribution functions (RDFs) of various atom type pairs:

(2) $$g\left(r\right)=4\pi r^{2}\rho dr,$$

where *ρ* represents number density of the unit cell, to directly and indirectly embed geometric and chemical information, respectively, into a vector or list [8,26,27]. RDF-based feature vector addresses cell periodicity issue by excluding interatomic distance contributions beyond a certain cutoff value. However, when all element combinations (~5000) are considered, RDF descriptors can easily become enormously large in dimensionality and be padded in large portion with zeros that do not contribute to learning. One recently reported representation shows good promise for solids and molecules and uses a kernel function *K* to compare, say between two solids *A* and *B*, based on smooth overlap of atomic positions [28]:

(3) $$K\left(A,B\right)=\sum_{i\varepsilon A,j\varepsilon B}P_{\mathrm{ij}}k\left(x_{i},x_{j}\right),$$

where *x*_*i*_, *x*_*j*_ relates to atom-centered environments and $P_{\mathrm{ij}}=\frac{1}{N_{A}N_{B}}$. Another representation attempted to include electronic structure information by binning energy diagrams of high symmetry points of a crystal’s Brillouin zone and binning of DOS data [16]. Truncated bispectrum approach was introduced to generate descriptors based on projected atomic density information onto a 4-D unit sphere surface for one-to-one representation of atomic neighborhoods [29]:

(4) $$B_{J_{1},J_{2},J}=\sum_{m_{1}^{{}^{\prime }},m_{1}=-J_{1}}^{J_{1}}\sum_{m_{2}^{{}^{\prime }},m_{m}=-J_{2}}^{J}\sum_{m^{{}^{\prime }},m=-J}^{J}{\left(c_{m^{{}^{\prime }}m}^{J}\right)}^{*}C_{J_{1}m_{1}Jm_{2}}^{\mathrm{Jm}}\times C_{J_{1}m^{{}^{\prime }}J_{2}m^{{}^{\prime }}}^{Jm^{{}^{\prime }}}c_{m_{1}^{{}^{\prime }}m_{1}}^{J_{1}}c_{m_{2}^{{}^{\prime }}m_{2}}^{J_{2}},$$

where $c_{m^{{}^{\prime }}m}^{J}$ are 4D spherical harmonics coefficients related to atom index *J* (atom index *I* is dropped for clarity in the equation), $C_{Jm_{1}j_{2}m_{2}}^{\mathrm{Jm}}$ are ordinary Clebsch–Gordan coefficients, and $J,J_{1},J_{2}\leq J_{\max}$ denote the spatial resolution limit defining the atomic neighborhood.

Another class of material representation was defined by decomposing infinite and periodic crystal structures into finite number of representative fragments based on rules of chemical bonding coordination sphere determined from Voronoi-tessellation cells of atom centers. The shape of a Voronoi cell is often described by three integer sets: $\left[F_{i}\right]$, $\left[V_{i}\right]$, $\left[E_{i}\right]$, where *F*, *V*, *E* are the number of *i*-edged faces, number of vertices, and number of edges, respectively, and $i\in \left[3,4,5,\ldots \right]$ [30,31]. In one study, Voronoi cell information was combined with atomic/chemical labeling in order to define crystal substructures for applications in structure similarity metrics and crystal site prediction [32]. In yet another study, a feature vector was formulated with a similar idea, constructing at first infinite 3D periodic graphs of vertices *V* (atoms) and edges *E* (bonding):

(5) $$G=\left(V,E\right),$$

and then eliminating the problem on cell periodicity by breaking down the conceptually infinite graphs into unit-cell-relevant subgraph types from which finite-sized adjacency matrices were then determined:

(6) $$A_{\mathrm{IJ}}=\left\{\begin{array}{cc} 1\; & \text{if}\;I\;\text{and}\;J\;\text{are adjacent}\\ 0\; & \text{otherwise} \end{array}\right.,$$

where *A* is for adjacency matrix element encompassing all atom pairing *I* and *J* in one subgraph type [33]. In these schemes, it is obvious that: (i) both chemical/atomic and geometrical properties are always considered in an attempt to make the representation universal, (ii) atomic or chemical descriptors from the literature may be made suitable for defining the ‘chemical identity’ of materials, and (iii) Voronoi cell features may provide the criteria to describe the ‘structure identity’ of materials.

In this work, we further explored the use of Voronoi tessellation for differentiating crystal structures of inorganic materials. However, instead of utilizing Voronoi features as criteria for formulating crystal substructures or fragments for defining ‘structure identity’, we directly binned the Voronoi feature real values themselves and utilize the bin count information to construct general vector-form descriptors. For ‘chemical identity’, histogram from atomic/chemical property data was added into the overall material fingerprint. The approach was validated by performing supervised learning on DFT-calculated properties for inorganic materials: cohesive energy (CE), density (*d*), electronic band gap (BG), and decomposition energy (Ed). Our proposed scheme offers a simple, robust and cost-effective alternative for generating feature vector representations for a wide variety of crystalline materials.

## 2. Computational details

### 2.1. DFT calculation, material data-set, target properties

The material properties that were used for supervised learning are CE, *d*, BG, and Ed. For generating the material data-set, DFT calculations were performed with the VASP code which uses projected augmented wave (PAW) potentials [34,35]. The standard generalized gradient approximation (GGA) with Perdew–Burke–Ernzerhof parametrization for solids (PBEsol) was applied to describe exchange correlation effects [36]. Under spin-polarized condition, structure geometry optimization was carried out with a cutoff of 500 eV in kinetic energy and this led to a convergence upper bound of 1 meV per formula unit in the total energy. *K*-point mesh was set to ≥1000 and with Monkhorst-Pack grids.

We focused our search space to Li-containing compounds owing to their importance as fast ionic conductors for batteries. Structure coordinate data were collected from the Inorganic Crystal Structure Database (ICSD) [37]. Separate 1000 data-sets were taken as well from Materials Project [1], for analyzing the effect of training data-set size on prediction performance. Transition metal elements were excluded to generate a rather homogeneous data-set avoiding entries that may contribute large uncertainties in error measures related BG calculation (usually underestimated by DFT). Duplicates and unphysical structures were removed to form the model building and test sets. Additional information is available in Table S1 (ICSD-based DFT data) and S2 (material ID of Materials Project data) of Supporting Information.

To determine CE, the energy of atoms in the crystal and the energy of corresponding free atoms need to be calculated from DFT:

(7) $$\text{CE}=E_{\text{crystal}}-\sum_{i=1}^{N_{\text{atom types}}}n_{i}E_{i},$$

where $E_{\text{crystal}}$ is the total energy of the compound, $N_{\text{atom types}}$ is the number of atom types, $n_{i}$ is the number of atoms of type *i*, and $E_{i}$ is the total energy of an atom type *i* (calculated by placing a single atom in the middle of a 20 × 20 × 20 – Å simulation box. Standard DFT typically underestimates BG by as much as 50%, particularly for insulators and semiconductors [38]. This discrepancy is usually solved by introducing the Hubbard U extension [39,40] or the GW many-body scheme [41,42] but the computation is extremely expensive, especially with respect to system size. Also, calculation for direct BG requires high k-point sampling and stricter energy convergence criterion. In this work, we considered the energy difference between the valence band top and conduction band bottom for DFT-BG estimation.

### 2.2. Formulation of generalized vector-form material descriptors

We took inspiration from the idea of binning and the frequency of occurrence for material-related features to generate ‘histograms of features’. We concatenated the histograms from atomic and geometric information around atom centers and use it to define a generalized input vector representation for any given material *x*:

(8) $$x={\left[h_{i}\right]}_{c}^{k_{1}}⌢{\left[h_{j}\right]}_{s}^{k_{2}},$$

where ${\left[\cdot \right]}_{c}$ and ${\left[\cdot \right]}_{s}$ are histograms for the ‘chemical’ and ‘structure’ identities of the material, respectively, joined together by a concatenation operator ⌢, *h*_*i*_ is the atomic feature count at bin *i* where $i\in \left[1,2,\ldots ,k_{1}\right]$ (*k*_1_ total bins), *h*_*j*_ is the local geometric feature count at bin *j* where $j\in \left[1,2,\ldots ,k_{2}\right]$ (*k*_2_ total bins).

**Figure 1.**
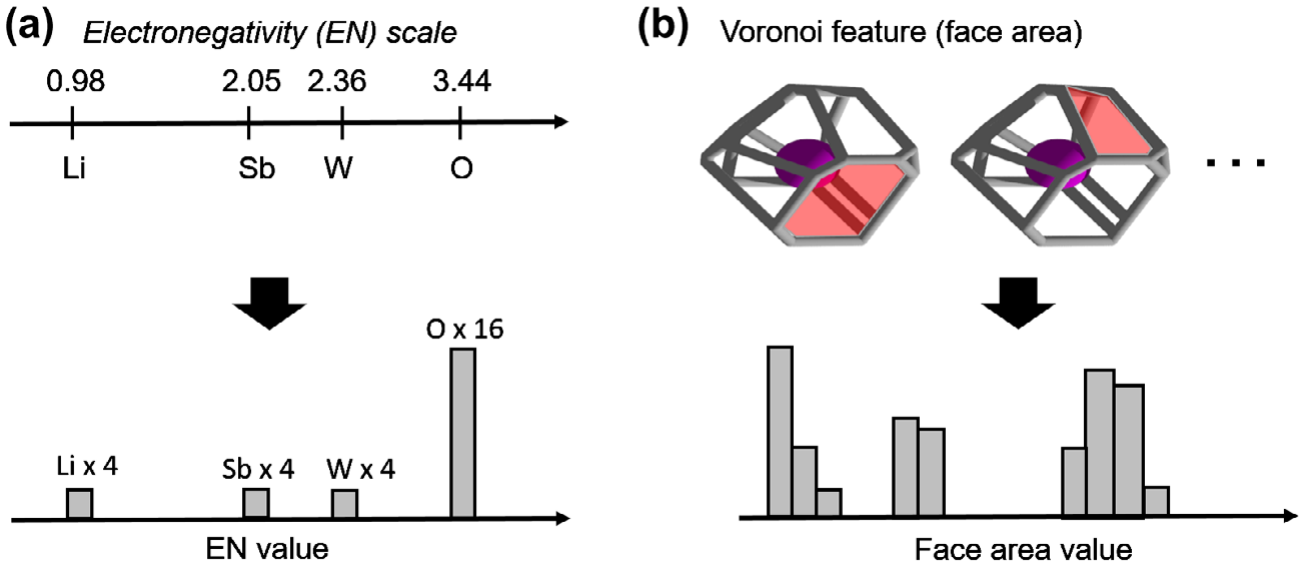
Example of feature binning procedure for (a) electronegativity and (b) Voronoi feature face area (in red) in *Pbcn* LiSbWO_6_ with 4 Li, 4 Sb, 4 W, and 16 O atoms in the unit cell.

Note: Central atom for the Voronoi cell in (b) is shown in magenta.

For ${\left[\cdot \right]}_{c}$, we chose the atomic property based on Pauling’s electronegativity of elements (EN) [43]. The reason for this choice is that it allows for defining chemical bonding in crystalline solids on a continuum or scale, whereby bonds can be mapped as strongly ionic on one extreme, strongly covalent on the other, or somewhere in between (i.e. a combination of both ionic and covalent character). Other atomic-related properties can also be considered and added as well such as electron affinity, atomization enthalpies, vaporization enthalpies, fusion enthalpies, melting point, atomic mass, atomic number, number of valence electrons, ionization potentials, covalent radii, ionic radii [44], and element periodic group number. However, it should be pointed out that further increasing the dimensionality of the material representation would also require an appropriate increase in the size of training set so as to reduce the risk of optimistic and biased evaluations on machine model performance [45]. In the present work, we ensure to mitigate the latter by performing subsampling (of training set and feature set), regularization, and cross-validation. Meanwhile, ${\left[\cdot \right]}_{s}$ was derived from Voronoi-based numerical feature values around each atoms in a crystal structure [46]. Figure 1 illustrates the proposed concept for generating atomic- and geometric-based binned features. Several local Voronoi features were collected such as number of vertices, number of edges, number of faces, face area, number of bounding edges per face, and Voronoi cell volume. Subsequently, ${\left[\cdot \right]}_{s}$ is itself constructed from concatenation of a series of histograms:

(9) $${\left[h_{j}\right]}_{s}^{k_{2}}={\left[h_{j}\right]}_{s,f_{1}}^{k_{2,1}}⌢{\left[h_{j}\right]}_{s,f_{2}}^{k_{2,2}}⌢{\left[h_{j}\right]}_{s,f_{3}}^{k_{2,3}}⌢\ldots ,$$

where ${\left[\cdot \right]}_{s,f_{1}}$, ${\left[\cdot \right]}_{s,f_{2}}$, and ${\left[\cdot \right]}_{s,f_{3}}$, and so on are histograms from Voronoi feature type 1, Voronoi feature type 2, and Voronoi feature type 3, and so on, respectively. Voronoi features that were included in Equation (9) are displayed in Table 1. Each Voronoi histograms were then bin-wise averaged by the total number of atoms in the original unit cell:

(10) $${\left[h_{j}\right]}_{s}^{k}=\frac{1}{N_{\text{atoms}}}\sum_{i=1}^{N_{\text{atoms}}}{\left[h_{j}\right]}_{s,i}^{k}$$

**Table 1.** Applied smoothing parameters for various histograms.

Feature	Symbol	Histogram range	Number of bins	Smoothing factor
Electronegativity	EN	0 < EN < 5	50	2
#Voronoi cell edges*	*a*	0 < *v* < 70	40	4
Area per Voronoi cell face*	*f*	0 < *e* < 90	50	4
#edges per Voronoi cell face*	*g*	0 < *f* < 50	30	4
#Voronoi cell faces*	*s*	0 < *a* < 15	18	1
Voronoi cell volume*	*v*	0 < *b* < 20	20	1
#Voronoi cell vertices*	*w*	0 < *V* < 25	25	1
RDF	RDF	0 < RDF < 6 Å	20	0.2 Å

* Features extracted around each central atom in a unit cell.

As there are several Voronoi feature types, we allow the machine learning models to determine naturally during the model building step which among the features are predictive for the target properties.

**Figure 2.**
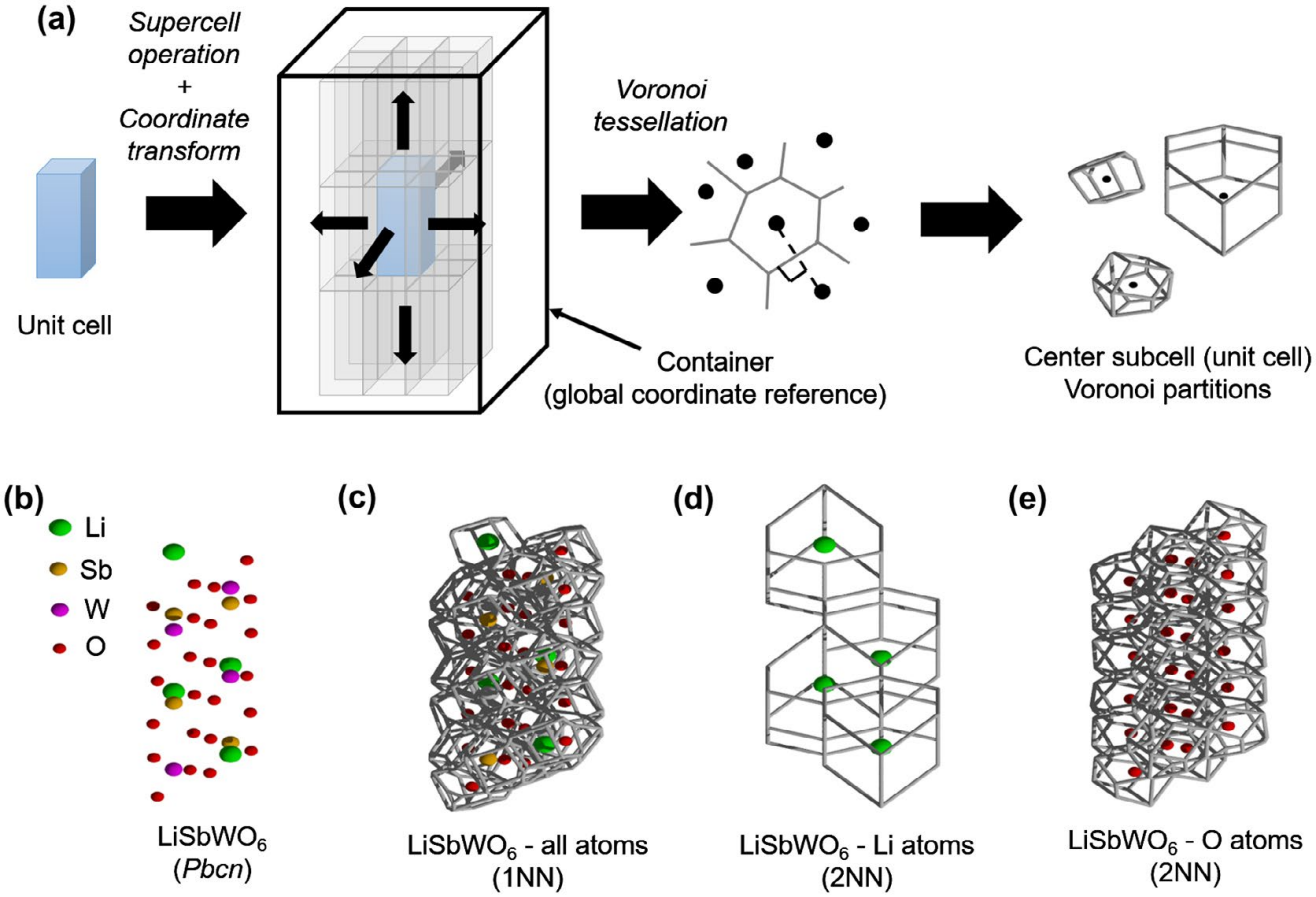
(a) Schematic workflow for the extraction procedure implemented for Voronoi tessellation of crystal structures, (b) Test case crystal structure *Pbcn* LiSbWO_6_ (green spheres are Li atoms, brown spheres are Sb atoms, magenta spheres are W atoms, and red spheres are O atoms). Voronoi tessellation for LiSbWO_6_ (c) with all atoms considered (1st nearest-neighbor (NN) information), (d) with Li atoms only (2NN information), and (e) with O atoms only (2NN information).

Note: Voronoi atom centers and Voronoi edges are shown in black and gray, respectively.

Given a structure coordinate data, the collection of features started from a given unit cell for input in 3D-Voronoi tessellation. To avoid the inclusion of artificial Voronoi shapes forming near the boundary wall, the unit cell was first expanded into a supercell (e.g. 3 × 3 × 3) as shown in Figure 2(a). The atomic coordinates were then transformed with respect to a larger container box of arbitrary size (shown as a rectangular parallelepiped). This container box served as a global reference for the coordinates, that is, a given atom coordinate $\vec{r}$ with supercell basis vectors $\vec{a}$, $\vec{b}$, $\vec{c}$ was transformed into the container basis vectors $\bar{a^{\prime }}$, ${\vec{b}}^{{}^{\prime }}$, ${\vec{c}}^{{}^{\prime }}$ as ${\vec{r}}^{{}^{\prime }}$:

(11) $${\vec{a}}^{{}^{\prime }}=s_{11}^{{}^{\prime }}\vec{a}+s_{12}^{{}^{\prime }}\vec{b}+s_{13}^{{}^{\prime }}\vec{c},$$

(12) $${\vec{b}}^{{}^{\prime }}=s_{21}^{{}^{\prime }}\vec{a}+s_{22}^{{}^{\prime }}\vec{b}+s_{23}^{{}^{\prime }}\vec{c},$$

(13) $${\vec{c}}^{{}^{\prime }}=s_{31}^{{}^{\prime }}\vec{a}+y_{32}^{{}^{\prime }}\vec{b}+s_{33}^{{}^{\prime }}\vec{c},$$

where the matrix coefficients $s_{\mathrm{ij}}^{{}^{\prime }}$ signifies changes in length, orientation, or both of basis vectors $\vec{a}$, $\vec{b}$, $\vec{c}$ in the new basis vectors ${\vec{a}}^{{}^{\prime }}$, ${\vec{b}}^{{}^{\prime }}$, ${\vec{c}}^{{}^{\prime }}$. Voronoi-feature real values were then extracted from atoms bounded only within the center subcell unit.

In general, the generated Voronoi-based histograms contain first-nearest-neighbor (1NN) information, noting that Voronoi facets appear between 1NN atoms joined together by lines bisected by planes perpendicularly. In order to explicitly include 2NN information, partitioning was performed as well for cation and anion subset coordinates of the original unit cell. An example is shown Figure 2(b)–(e) for a test case crystal structure *Pbcn* LiSbWO_6_ [47]. The overall input vector formed by combining EN and Voronoi histograms are hereafter called EN+VORO feature vector for simplicity. Voronoi tessellation was carried out using the Voro++ library [48].

### 2.3. Supervised learning details

In this work we focused on four material properties for building machine models of the form *y* = *f*(*x*): CE, d, BG and Ed. Given that these targets are continuous data, the supervised learning task essentially falls under a regression approach wherein an error loss function is minimized:

(14) $$\min_{f}\sum_{i=1}^{n}L\left(f\left(x_{i}\right),y_{i}\right)+\lambda g\left(f\right),$$

where the first and second term are the loss and regularization function, respectively. The loss function is assumed with a squared form:

(15) $$L\left(f\left(x\right),y\right)=\frac{1}{2}{\left(y-f\left(x\right)\right)}^{2},$$

Meanwhile, $g\left(f\right)$ describes the measure of complexity or roughness of *f* and λ is a model hyperparameter that is tuned during model training and validation.

There are many regressors that can be used to evaluate the predictive performance of material descriptors and representations for numerical target material properties, each of them can lead to varying levels of out-of-sample error [49]. In this work, two distinct nonlinear regressors were chosen, the first one takes a predetermined predictor form while the second one has a form that has to be supplied by the data itself: gradient boosting regression (GBR) with decision-tree base learners and epsilon (ε)-support vector regression (ε-SVR) with radial basis function (RBF) kernel [50,51].

In tree-based GBR, the *additive* expansion of *f*(***x***) is given by the form:

(16) $$f\left(x\right)=\sum_{m=1}^{M}\beta_{m}h\left(x;a_{m}\right),$$

where *β*_*m*_ represents the coefficient of base tree *h* with hyperparameter set a. Base tree learners are fitted to residuals $y_{i}-f\left(x_{i}\right)$, instead of *y*_*i*_, in a forward stage-wise manner:

(17) $$\Theta =\sum_{i=1}^{\ell }\frac{1}{2}{\left(y_{i}-f\left(x_{i}\right)\right)}^{2},$$

where *Θ* is the summed residuals for all *ℓ* training data points. Taking the derivative of *Θ* with respect to $f\left(x_{i}\right)$ leads to the interpretation for residuals as negative gradients:

(18) $$y_{i}-f\left(x_{i}\right)=-\frac{\partial \Theta }{\partial f\left(x_{i}\right)}.$$

Equation (18) enables the use of steepest-descent approach for improving prediction related to $f\left(x\right)$. Performing a stage-wise strategy of adding the *m*th base tree will update *β* and a:

(19) $$\left(\beta_{m},a_{m}\right)=\underset{\beta ,a}{\text{argmin}}\sum_{i=1}^{N}\Theta \left(y_{i},f_{m-1}\left(x_{i}\right)+\beta h\left(x_{i};a\right)\right).$$

Equation (18) then leads to the *m*th update of $f\left(x\right)$:

(20) $$f_{m}\left(x\right)=f_{m-1}\left(x\right)+\beta_{m}h\left(x;a_{m}\right).$$

In ε-SVR, the starting point is the familiar expression for the case of linear functions:

(21) $$f\left(x\right)=w,x+b,$$

where 〈⋅,⋅〉 indicates dot product operation in ***x***, w∈x, and b∈R. The goal is to find f(x) that has the largest deviation ε from *y*_*i*_, using all training data, while at the same time aiming to obtain a flat f(x) as much as possible. To extend SVR to nonlinear functions, the kernel trick is employed such as the RBF:

(22) $$k\left(x_{i},x_{j}\right)=\text{exp}\left(-\frac{{\left|\right|x_{i}-x_{j}\left|\right|}^{2}}{2\sigma^{2}}\right),$$

where *σ* is a free parameter.

Data splitting for model training and validation (80% of total data-set), and testing (20% of total data-set) was instantiated randomly for 100 times at each hyperparameter setting implemented (for both GBR and SVR fitting). Model selection was based on 10-fold cross validation (CV), with the final model being selected based on the lowest test data-set error (i.e. using only excluded data points from training and validation steps). Hyperparameters for both machine models were optimized by exhaustive cross-validated search over specified range of parameters (see Table 2). All fitting tasks were performed using the scikit-learn machine learning toolkit [52]. We used the grand average RMSE as a fitting stopping criterion for hyperparameter tuning and then picked the final model instance based on:

**Table 2.** Fitting conditions employed for GBR and SVR models.

Model	Hyperparameter	Condition
GBR	Base tree depth	3
	#Splitting per node	2
	#Base trees, *n*_trees_*	*n*_trees_ ∊ [10, 420]
	#Subsample features for node splitting	$\sqrt{h_{\text{total}}}$, where $h_{\text{total}}$ is the total number of bin features
	#Subsample training data	0.8
	Learning rate	0.1
SVR	Kernel	RBF
	Kernel coefficient	$1/h_{\text{total}}$
	*f* flatness – deviation tolerance tradeoff, C*	$C\in \left[1000,4000\right],\Delta C=100$
	Deviation cutoff, ε*	$\varepsilon \in \left[0.01,6.0\right],\Delta \varepsilon =0.01$

* Hyperparameters optimized by exhaustive cross-validated search over specified range of parameters.

(23) $$\underset{t\in \left[1,2,3,\ldots ,100\right]}{\text{argmin}}\left[e_{t}\right],$$

where *e*_*t*_ is the test error for fitting instance *t* in one hyperparameter setting.

Table 2 shows the model conditions implemented for GBR and SVR fitting. For comparison, CE and BG were also fitted using RDF feature vector consisting of histograms generated from all-atom, cation–cation, anion–anion, Li–Li, O–O, and Li–O interatomic pair contributions.

## 3. Results and discussion

### 3.1. Predictive power of formulated vector-form descriptors

**Figure 3.**
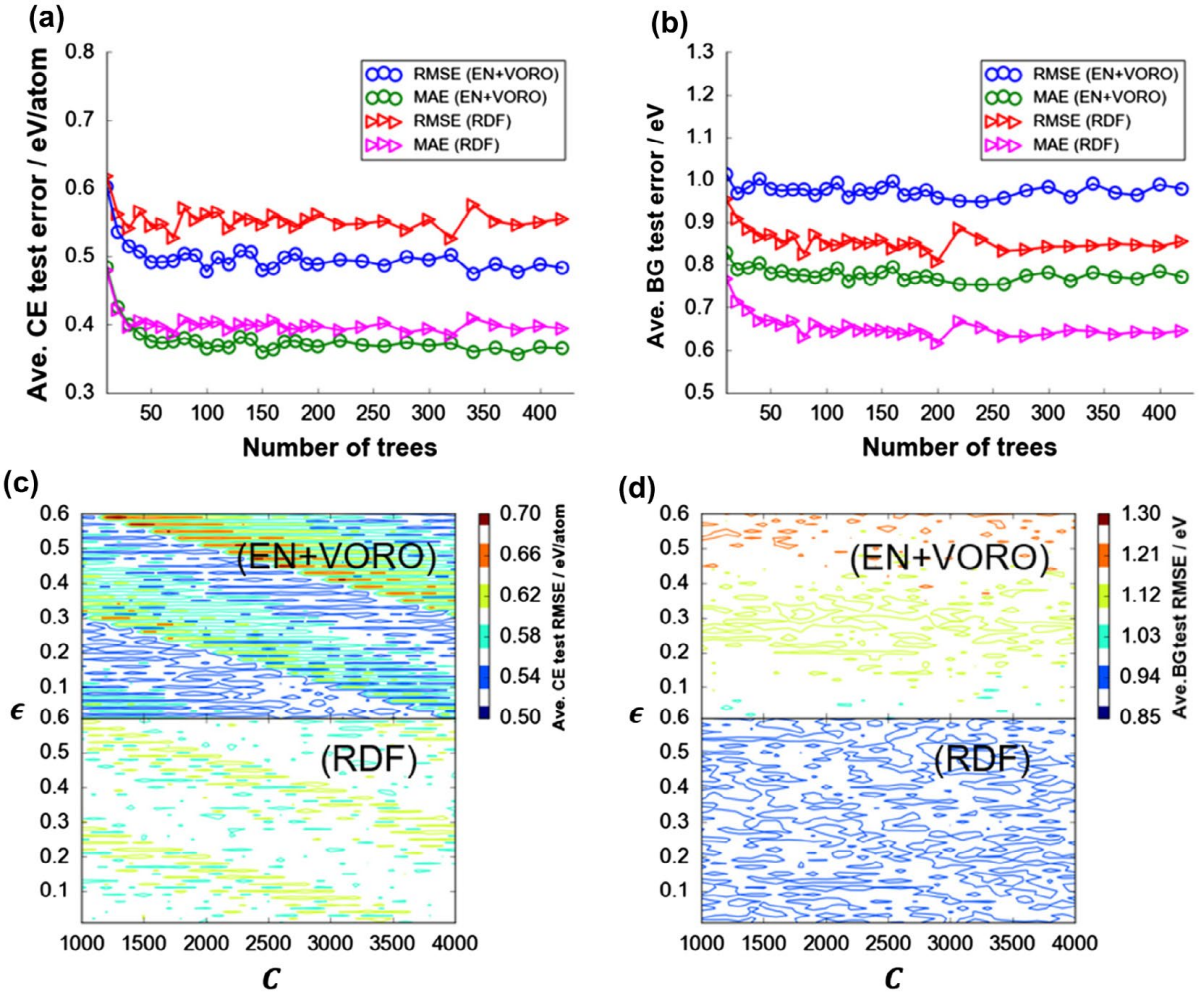
Test data-set grand average errors for DFT-CE and DFT-BG by (a), (c) GBR (total: 420 trees/data split × 100 data splits = 4200 fitting instances for CE and BG, respectively) and (b), (d) SVR fitting. Test data-set error surface with respect to hyperparameter combination (*C* and ε) for SVR fitting for (c) DFT-CE and (d) DFT-BG; a total of 18,600 regularly-spaced hyperparameter coordinate sets (or fitting instances) each, respectively.

Figure 3 shows the post-validation step evaluation results of the final models for DFT-CE and DFT-BG predictions with using ICSD-based test data-sets (see Table S1 in Supporting Information). Grand average RMSE_test_ and MAE_test_ (from 100 random data-set splitting) tends to converge after 50 and 70 base tree learners, respectively, were sequentially added. Note that the present RDF feature vectors include both chemical composition (implicitly) and crystal structure information, while EN or VORO feature vectors contains as well composition (explicitly) or crystal structure information, respectively, in this study. The errors do not increase even up to 420 trees owing to the subsampling routine implemented during the training step (i.e. 20% of the training data-set were always left out during the training step). For DFT-CE, EN+VORO outperforms RDF on the average by ∆RMSE_ave_ = 0.053 eV (∆MAE_ave_ = 0.025 eV). However, for DFT-BG prediction (Figure 3(b)), the reverse is true, with RDF predicting better than EN+VORO by ∆RMSE_ave_ = 0.110 eV (∆MAE_ave_ = 0.108 eV). On the other hand, for DFT-CE prediction by SVR, the resulting error surface with respect to *C* and ε hyperparameters is apparently stratified with peaks and trough for both EN+VORO and RDF feature vectors (Figure 3(c)). The error magnitude comparison is consistent with GBR results, that is, EN+VORO predicts more accurately than RDF by ∆RMSE_ave_ = 0.043 eV/atom (∆MAE_ave_ = 0.030 eV/atom) for DFT-CE. For DFT-BG, RDF slightly predicts better than EN+VORO by ∆RMSE_ave_ = 0.110 eV (∆MAE_ave_ = 0.096 eV). Error for EN+VORO is relatively more sensitive than RDF with respect to increasing ε (Figure 3(d) top) but is independent of *C*. RDF also has a narrower range for RMSE_ave_ than EN+VORO (0.116 eV vs. 0.284 eV, respectively). The optimal SVR hyperparameter settings are ${\left\{C=1900.0,\varepsilon \;=\;0.490\right\}}_{\text{DFT-CE}}$ and ${\left\{C=2600.0,\varepsilon \;=\;0.390\right\}}_{\text{DFT-BG}}$.

**Figure 4.**
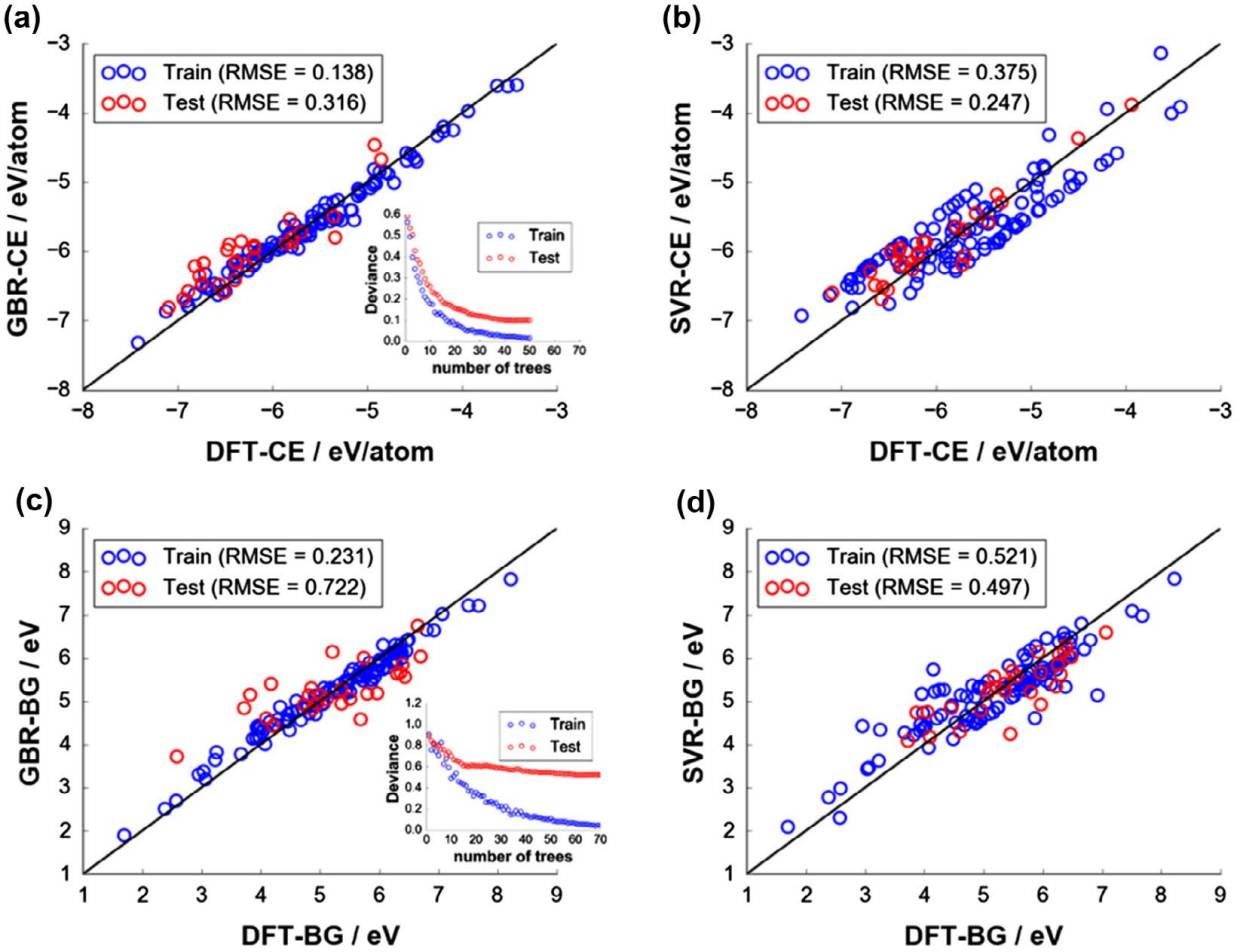
Fitting quality of final models: (a) CE with GBR, (b) CE with SVR, (c) BG with GBR, and (d) BG with SVR. Insets in (a) and (c) show deviance (error residuals) plots terminating at the optimal number of base tree learners for GBR fitting (50 trees for CE and 70 trees for BG, respectively). Optimal hyperparameters (*C* and ε) for SVR fitting are indicated in (b) and (d).

Figure 4 displays the fitting quality of the final models for DFT-CE and DFT-BG prediction with EN+VORO and RDF descriptors by GBR and SVR fitting using ICSD-based DFT data-sets with 140 compounds. For DFT-CE, EN+VORO provides better prediction than RDF with GBR fitting by ∆RMSE_test_ = 21.0 meV/atom (∆MAE_test_ = 23.0 meV/atom) and with SVR fitting by ∆RMSE_test_ = 19.0 meV/atom (∆MAE_test_ = 6.0 meV/atom). For DFT-BG, EN+VORO underperforms relative to RDF: ∆RMSE_test_ = 0.164 eV (∆MAE_test_ = 0.154 eV and ∆RMSE_test_ = 0.089 eV (∆MAE_test_ = 0.058 eV) by GBR and SVR approach, respectively. Insets in Figure 4(a) and (c) show the converged deviance (error residuals) for training and test sets for GBR fitting, stopping at the optimal number of trees of 50 and 70 trees for DFT-CE and DFT-BG, respectively). Despite the slightly larger variance in errors for EN+VORO for DFT-BG prediction, its coefficient of determination test is still reasonable (*R* ^2^ = 0.70). A summary of test set prediction metrics of the final models are listed in Table 3. Improvement on generalization for EN+VORO may be realized by increasing the size of training data, given that it may have a larger learning capacity than RDF, as suggested by the larger error difference between training and test data-sets for the former (e.g. 0.673 eV vs. 0.591 eV, respectively, for ΔRMSE_train-test_ for DFT-CE by GBR with 70 trees) [53].

**Table 3.** Comparison of predictive accuracy between EN+VORO and RDF-based final machine models with ICSD-based DFT calculated data-set (140 compounds).

Target property	Feature vector	Model	Test set RMSE	Test set MAE
DFT-CE (eV/atom)	EN+VORO	GBR	0.316	0.258
		SVR	0.247	0.196
	RDF	GBR	0.337	0.281
		SVR	0.266	0.202
DFT-BG (eV)	EN+VORO	GBR	0.722	0.587
		SVR	0.497	0.387
	RDF	GBR	0.558	0.433
		SVR	0.408	0.329

**Figure 5.**
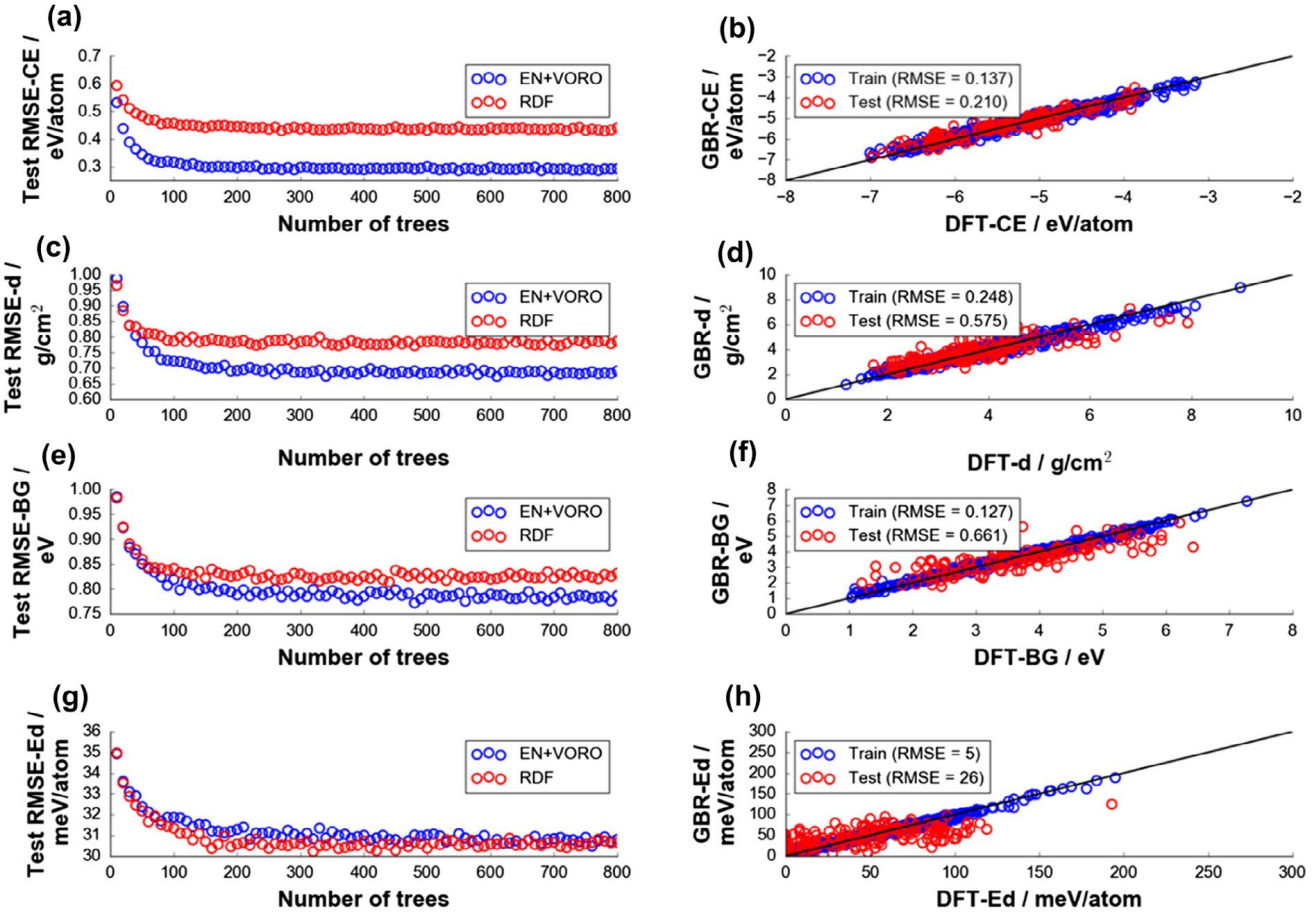
Test data-set grand average errors (from 100 random data splitting) for (a) DFT-CE, (c) DFT density (DFT-d), (e) DFT-BG, and (g) DFT decomposition energy (DFT-Ed) by GBR fitting. Final models have (b) 120, (d) 160, (f) 300, (h) 430 trees, respectively.

Notes: Data-sets are comprised with 1000 Li-containing oxides taken from Materials Project (see Table S2 of Supporting Information for the materials ID list) [1].

To further check the predictive performance of EN+VORO, fitting was also performed by GBR using a larger DFT data-set of 1000 Li-containing oxide compounds randomly extracted from Materials Project [1]. Properties such as DFT-CE, density (DFT-d), DFT-BG, and decomposition energy (DFT-Ed) were chosen for supervised learning using GBR approach. Results from hyperparameter tuning with respect to the number of boosting trees are shown in the left column of Figure 5. Plots showed a typical error convergence behavior. Fitting stopping criteria of 120, 160, 300, and 430 boosting trees were chosen with respect to RMSE_ave,test_ for DFT-CE (0.305 eV/atom), DFT-d (0.701 g/cm^3^), DFT-BG (0.792 eV), and DFT-Ed (31 meV/atom), respectively (final models shown in the right column of Figure 5). RMSE_ave,test_ for RDF is noted to be higher for DFT-CE (0.451 eV/atom), DFT-d (0.794 g/cm^3^) and DFT-BG (0.830 eV), while it has comparable error for DFT-Ed (31.0 meV/atom) vs. EN+VORO. We note that the result on DFT-BG here is opposite to what was observed using the relatively modest data-set size from ICSD as shown in Figure 4(b); RMSE_test_ by EN+VORO is now decreased from 0.722 to 0.661 eV. This means that the machine models fitted with EN+VORO and larger data-set size show less contribution from interpreting data noise as signal during model training step (variance is now lower). Based on a computational study by Morales-Garcia et al. [54], a linear relationship between DFT-GGA and the more accurate GW method for BG of oxides can be drawn, with a systematic underestimation error of about +0.9 eV by the former. Since this +0.9 eV offset is only a systematic shift above the ideal ‘BG_GW_ = BG_DFT-GGA_’ line, it is expected that EN+VORO would still be superior over RDF if GW method is used instead of present DFT-GGA method. However, we caution that this relationship is reasonable only for oxide-type materials. On the other hand, RMSE_test_ for DFT-BG by RDF has increased from 0.558 to 0.728 eV with the larger data-set, reflecting a lower learning capacity of RDF compared to EN+VORO. Final models for EN+VORO and RDF are summarized in Table 4, including SVR fitting results.

**Table 4.** Comparison of predictive accuracy between EN+VORO- and RDF-based final machine models using Materials Project data-set (1000 compounds).

Target property	Feature vector	Model	RMSE_test_	MAE_test_
DFT-CE (eV/atom)	EN+VORO	GBR	0.210	0.155
		SVR	0.274	0.196
	RDF	GBR	0.381	0.284
		SVR	0.410	0.318
DFT-d (g/cm^3^)	EN+VORO	GBR	0.575	0.436
		SVR	0.532	0.404
	RDF	GBR	0.653	0.479
		SVR	0.603	0.444
DFT-BG (eV)	EN+VORO	GBR	0.661	0.515
		SVR	0.668	0.541
	RDF	GBR	0.728	0.579
		SVR	0.730	0.590
DFT-Ed (meV/atom)	EN+VORO	GBR	26.0	22.0
		SVR	31.0	26.0
	RDF	GBR	27.0	21.0
		SVR	30.0	25.0

Above promising results validate our approach of directly binning Voronoi-based real values for descriptor vector construction for crystalline solids. It can be argued that the reason why EN+VORO generally outperforms RDF is that the former contains richer information (data signal over noise ratio is larger) and a larger learning capacity owing to the explicit inclusion of atomic/elemental features and the Voronoi features that are primarily generated by high-symmetry directions between lattice points of atom centers. That is why Voronoi-based information can be effectively used as criteria to reasonably define cutoffs for local atom coordination and environments (see Refs. [32,33]). In the case of RDF, atomic/elemental features are implicit only to the atom type pairings used for generating distance features and the 3D spatial information in terms of the relative positions of more than two atoms is mostly lost as well.

### 3.2. Feature importance analysis from GBR modeling

**Figure 6.**
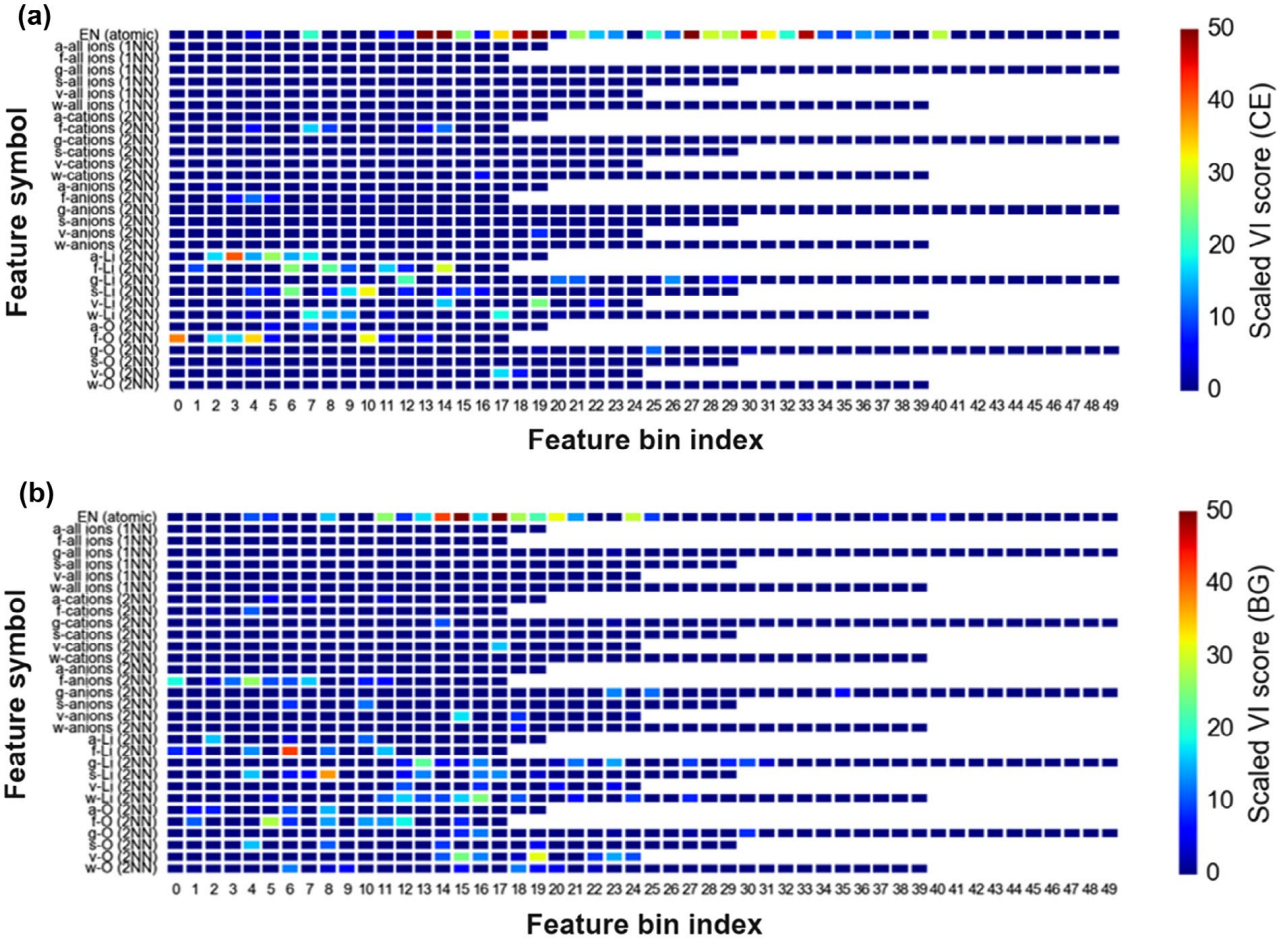
Scaled variable importance (VI) plot of bin features from the final GBR models for (a) DFT-CE and (b) DFT-BG.

Notes: Horizontal axis denotes bin index number of increasing real value feature from left to right. Vertical axis shows the labels of the subset histogram features (see Table 1 for description). VI scores are scaled to 100 while colorbar maximum is set only to 50 to enhance visual distinction among features.

Figure 6 shows the variable importance (VI) score plots (color gradient scaled to 100) of various feature bins constituting EN+VORO descriptors. The horizontal axis denotes the histogram bin index number arranged in increasing order from left to right with respect to the subset feature real value. The vertical axis indicates the description for each subset features.

It is apparent for DFT-CE that the atomic subset histogram bins are significantly contributing towards prediction (VI scores up to 50 and higher). Meanwhile, all Voronoi-based 1NN bin features do not contribute significantly. Voronoi-based 2NN bin features for Voronoi face area and volume from all-cations and all-anions are noted to contribute towards prediction (*f*-cations, VI score = ~20; *f*-anions, VI score = ~10; *v*-anions, VI score = ~10). All 2NN Li-cation Voronoi cell features contribute significantly (VI score reaching as much as ~40). For the O-anions features, face area and cell volume are descriptive (VI score = ~30).

For DFT-BG predictions, several bins from the EN histogram also show high VI scores (up to 50 and higher). Again, no notable 1NN bin features appear to be meaningful. Similar with DFT-CE, 2NN bins from face area and volume from all-cations and all-anions are contributing towards DFT-BG prediction (VI score up to ~25). All 2NN features for Li-cations and most of O-anions contribute as well (VI score up to ~40).

**Figure 7.**
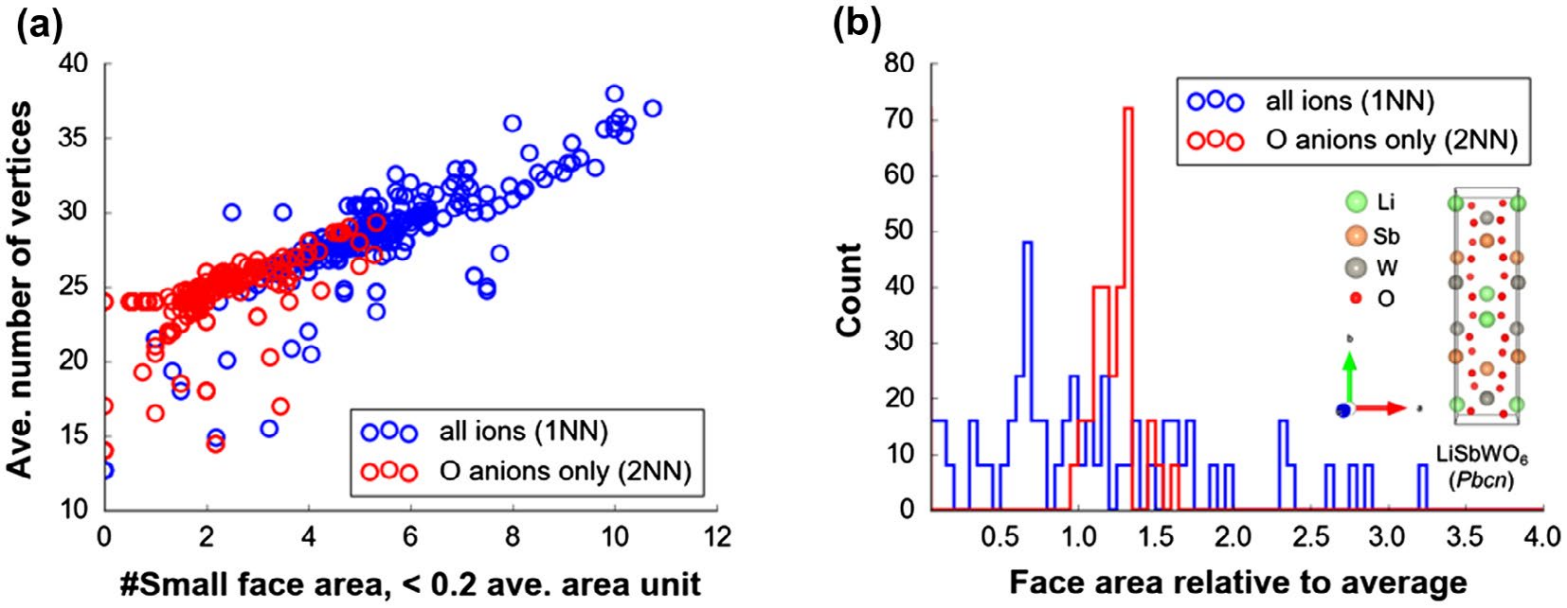
(a) Average number of vertices vs. counted average face area <0.2 from Voronoi tessellations of atom centers, (b) histogram for face areas relative to average per Voronoi cell for *Pbcn* LiSbWO_6_ crystal structure.

Based from above observations on feature importance, 2NN Voronoi features are consistently determined as good predictors in comparison to their 1NN counterparts. EN features are also confirmed to be good predictors. The low VI scores of 1NN Voronoi features may be explained by fluctuation effect from artificial increase of histogram bin counts because of Voronoi cell faces that are actually not physically meaningful (i.e. faces that are not formed by nearest-neighbor information or formation of chemical bonding), causing ambiguity when differentiating crystal structures [55]. This is supported by the analysis of Figure 7(a) which displays the *linear* relationship between number of vertices vs. counted small face areas (<0.2 average face area unit) for DFT-optimized structures related to Figures 3 and 4. It is clear that 1NN features tend to have smaller areas with large number of vertices than 2NN features. Intuitively, this causes the resulting bin counts to be spread out leading to a histogram feature vector with the primary peak broadened and shifted towards the low value region (see Figure 7(b)). In the case of 2NN features, the histogram shape shows a relatively sharp primary peak.

## 4. Conclusions

A generalized histogram-based representation for crystalline solids, which is invariant to atom ordering, cell periodicity, and number of atoms, was constructed by binning atomic/chemical property data and Voronoi-tessellation features. The formulated material descriptor vector showed a better predictive power and generalization performance relative to RDF-based descriptor vector for DFT-calculated properties such as cohesive energy, density, band gap, and decomposition energy. From variable importance analysis, it was confirmed that binned atomic/chemical property and 2NN Voronoi cell geometric property data are good predictors.

## Disclosure statement

No potential conflict of interest was reported by the authors.

## Supplemental data

The supplemental data for this article can be accessed at https://doi.org/10.1080/14686996.2018.1439253

## Funding

This work was supported by ‘Materials research by Information Integration’ Initiative (MI^2^I); the Development Program of the Japan Science and Technology Agency (JST) under ‘Advanced Materials Informatics through Comprehensive Integration among Theoretical, Experimental, Computational, and Data-centric Sciences’ research area and by ‘Elements Strategy Initiative to Form Core Research Center’ (Since 2012); Ministry of Education Culture, Sports, Science and Technology (MEXT); JST Precursory Research for Embryonic Science and Technology (PRESTO) program for the financial support.

## Supplementary Material

Supporting_information.docx
