# Supplementary material for: A general representation scheme for crystalline solids based on Voronoi-tessellation real feature values and atomic property data
Source: Sci Technol Adv Mater. 2018 Mar 19;19(1):231–42. doi: 10.1080/14686996.2018.1439253 (PMC5917445; doi:10.1080/14686996.2018.1439253)
Supplement: Supporting_information.docx [file TSTA_A_1439253_SM2940.docx]

# Supporting information

## A General Representation Scheme for Crystalline Solids Based on Voronoi-Tesellation Real Feature Values and Atomic Property Data

R. Jalem^*^, M. Nakayama, Y. Noda, T. Le, I. Takeuchi, Y. Tateyama and H. Yamazaki

Table S1. DFT-calculated Li-containing oxides from Inorganic Crystal Structure Database (ICSD).

| ICSD # | Unit-cell chemical formula | #Atoms in the unit cell | DFT-CE / eV/atom | DFT-BG / eV |
| --- | --- | --- | --- | --- |
| 153807 | Li8O16S4 | 28 | -4.9271 | 6.6541 |
| 159530 | Al4Li4O24Si8 | 40 | -6.559 | 6.2388 |
| 160603 | B12Li4O56Y24 | 96 | -7.4172 | 4.7338 |
| 167517 | In4Li4O16Si4 | 28 | -5.7226 | 3.9797 |
| 168500 | B12Cl12Li2O16S8 | 50 | -4.7768 | 1.7001 |
| 200981 | B56Cl8Li40O100 | 204 | -6.4554 | 6.4845 |
| 201138 | Li4Mg4O16P4 | 28 | -5.7055 | 6.1386 |
| 241234 | B4Li6O12Sc2 | 24 | -6.3727 | 5.0188 |
| 34256 | B4Li4O8 | 16 | -6.3817 | 8.2239 |
| 45511 | Li4O8Y4 | 16 | -6.3654 | 5.2136 |
| 60935 | In2Li2O14P4 | 22 | -5.6025 | 5.0688 |
| 68475 | B14Li6O24 | 44 | -6.8148 | 6.3564 |
| 72101 | Ga4Ge4Li12O20 | 40 | -5.0944 | 4.0554 |
| 74860 | Al4Li4O48P16 | 72 | -5.9445 | 6.3843 |
| 79379 | Ca4Li4O26Si10 | 44 | -6.4766 | 5.4559 |
| 83831 | Li6O72P18Sn12 | 108 | -5.6498 | 3.9273 |
| 87987 | Cl2Ga6Li8O24Si6 | 46 | -5.6859 | 5.2383 |
| 100403 | Ge4Li8O12 | 24 | -5.0806 | 4.286 |
| 20434 | Al2B4Li6O12 | 24 | -6.191 | 6.3022 |
| 23723 | Li8O16S4 | 28 | -4.9661 | 6.8041 |
| 31050 | Ge8Li24O28 | 60 | -4.9396 | 4.7072 |
| 37060 | B4Li4O8 | 16 | -6.3085 | 5.8404 |
| 415201 | B12Li4O20 | 36 | -6.6457 | 4.9558 |
| 419852 | Cl12Li20O4 | 36 | -3.6285 | 4.0976 |
| 50420 | Li12O48P12Sc8 | 80 | -6.2074 | 4.9493 |
| 59243 | Li8O14P4 | 26 | -5.4493 | 6.4585 |
| 59936 | Cl4Li4O8 | 16 | -3.4222 | 2.5883 |
| 62481 | Ge4Li4O16Sc4 | 28 | -6.1075 | 4.4907 |
| 65125 | Ga18Li18O72Si18 | 126 | -5.8956 | 4.9583 |
| 67535 | B2Ge2Li2O8 | 14 | -6.0249 | 5.8012 |
| 71875 | B10Ba4Li2O20 | 36 | -6.8886 | 5.2356 |
| 83832 | Li2O24P6Sn4 | 36 | -5.6551 | 4.4549 |
| 85714 | Li12O36P12 | 60 | -5.5806 | 6.3018 |
| 92708 | Al18Li18O72Si18 | 126 | -6.4124 | 5.8691 |
| 180289 | Ba6Li12O24Si6 | 48 | -5.7915 | 5.0394 |
| 185703 | Al4Ge8Li4O24 | 40 | -5.7269 | 4.3322 |
| 22015 | Al3Li3O12Si3 | 21 | -6.3937 | 6.476 |
| 25752 | Li12O14Si4 | 30 | -5.4783 | 5.7272 |
| 28106 | B4Ge4Li4O16 | 28 | -5.8167 | 3.2349 |
| 34079 | Li4O16Si4Y4 | 28 | -6.7447 | 5.6899 |
| 50612 | Al4B4Li8O16 | 32 | -6.1348 | 5.4875 |
| 639886 | In4Li4O8 | 16 | -4.7167 | 3.0399 |
| 68429 | B16Li4O40Sc4Sr8 | 72 | -6.8819 | 4.8015 |
| 1180 | Li24O18Sn3 | 45 | -4.5002 | 4.8987 |
| 100402 | Li8O12Si4 | 24 | -5.7651 | 5.7827 |
| 16229 | Al2Li10O8 | 20 | -4.9222 | 5.8647 |
| 167518 | Ge4In4Li4O16 | 28 | -5.1373 | 3.2577 |
| 180557 | Li4O4 | 8 | -4.0915 | 3.0702 |
| 23815 | Al4Li4O8 | 16 | -5.9613 | 6.9249 |
| 24143 | Li16O16 | 32 | -3.9357 | 2.3869 |
| 261256 | B4Li6O12Sc2 | 24 | -6.3727 | 4.916 |
| 28178 | Ge8Li8O20 | 36 | -5.1755 | 3.9358 |
| 30295 | Al4Li4O24Si8 | 40 | -6.559 | 6.2586 |
| 34361 | Ge18Li8O40 | 66 | -5.3435 | 4.1779 |
| 415200 | B12Li4O20 | 36 | -6.5099 | 3.7249 |
| 62878 | In12Li18O72P18 | 120 | -5.4881 | 4.4515 |
| 77544 | Li4O16Sc4Si4 | 28 | -6.7091 | 4.8217 |
| 9105 | B4Li12O12 | 28 | -5.5137 | 5.7274 |
| 95972 | Li8Mg4O16Si4 | 32 | -5.711 | 5.3354 |
| 99503 | B4Ca2Li8O12 | 26 | -5.869 | 5.2502 |
| 162784 | Li4O48P16Y4 | 72 | -6.1109 | 6.4368 |
| 60948 | In8Li12O48P12 | 80 | -5.4868 | 4.6203 |
| 65025 | Li8Mg8O48S12 | 76 | -5.1619 | 6.3701 |
| 33716 | Ga20Li4O32 | 56 | -5.3259 | 4.2122 |
| 72486 | B16In4Li4O40Sr8 | 72 | -6.4943 | 4.4658 |
| 10257 | Li6O8P2 | 16 | -5.3577 | 7.6814 |
| 8222 | Li56O56Si14 | 126 | -5.3068 | 5.7842 |
| 424308 | B24Li4O46Sr8 | 82 | -6.9373 | 4.8154 |
| 36124 | Li4O8Sc4 | 16 | -6.4684 | 4.0771 |
| 67536 | B2Li2O8Si2 | 14 | -6.6086 | 7.5105 |
| 50957 | Al6Li18O58P16 | 98 | -5.8145 | 6.2247 |
| 66387 | Ca6Li6O24P6 | 42 | -5.9099 | 5.6791 |
| 98845 | Al2Li2O12Si4 | 20 | -6.5262 | 5.9791 |
| 65176 | Li16O12Si2 | 30 | -4.8682 | 5.5133 |
| 51314 | Al8B16Li8O40 | 72 | -6.7249 | 5.9682 |
| 279578 | Al4B20Li8O40 | 72 | -6.8161 | 6.2334 |
| 42697 | Al8Li40O32 | 80 | -4.9285 | 5.3666 |
| 79867 | Ca4Li16O32Si8Sr4 | 64 | -5.8373 | 5.4295 |
| 93087 | Ga4Li4O8 | 16 | -5.0975 | 5.5121 |
| 93015 | B54Li6O90Sr6 | 156 | -7.094 | 6.2745 |
| 91496 | Li2O14P4Sc2 | 22 | -6.2503 | 5.1134 |
| 173180 | Li8O4 | 12 | -4.1948 | 4.873 |
| 300010 | B32Li16O56 | 104 | -6.7419 | 6.2748 |
| 51630 | Li20O60P20 | 100 | -5.5825 | 6.3613 |
| 21053 | Li16O24Sn8 | 48 | -4.9074 | 3.8713 |
| 108886 | Li6O3 | 9 | -4.1948 | 5.9945 |
| 9082 | Ga8Li40O32 | 80 | -4.5832 | 4.5301 |
| 72100 | Ga4Li12O20Si4 | 40 | -5.5055 | 4.255 |
| 73218 | B4Ba4Li4O12 | 24 | -6.2044 | 4.9444 |
| 184292 | B12Li24O36Y4 | 76 | -6.0632 | 5.1828 |
| 39814 | Li16O28P8 | 52 | -5.4496 | 6.4978 |
| 20208 | Li12O16P4 | 32 | -5.3557 | 6.3202 |
| 51754 | Al2B4Li6O12 | 24 | -6.1919 | 6.29 |
| 19023 | Ca2Li4O8Si2 | 16 | -5.8561 | 6.1223 |
| 152065 | Ga4Li4O24Si8 | 40 | -6.1802 | 5.447 |
| 172184 | Ga6Li18O58P16 | 98 | -5.5736 | 5.5158 |
| 415199 | B12Li4O20 | 36 | -6.5756 | 2.958 |
| 66137 | Al4Li4O16Si4 | 28 | -6.3848 | 6.0089 |
| 15112 | In12Li36O36 | 84 | -4.4798 | 4.0026 |
| 98615 | Li8O8Si2 | 18 | -5.3114 | 5.68 |
| 99386 | B8Ca8Li8O24 | 48 | -6.2724 | 5.6901 |
| 184917 | La4Li4O48P16 | 72 | -6.1357 | 5.6206 |
| 72098 | Al4Ge4Li12O20 | 40 | -5.4409 | 4.9279 |
| 55178 | In4Li4O24Si8 | 40 | -6.0722 | 4.6036 |
| 94355 | B8In4Li12O24 | 48 | -5.795 | 4.1544 |
| 167334 | Li6O12Si3Sr3 | 24 | -5.8012 | 5.4664 |
| 93013 | B54Ba6Li6O90 | 156 | -7.1213 | 6.2477 |
| 68427 | Al6Cl2Li8O24Si6 | 46 | -6.1559 | 6.0047 |
| 15414 | Li8O20Si8 | 36 | -6.1442 | 5.9251 |
| 16408 | Al4Cl16Li4O24S12 | 60 | -4.2586 | 2.5769 |
| 19024 | Ca2Ge2Li4O8 | 16 | -5.3372 | 5.7433 |
| 16486 | B4Cl16Li4O48S16 | 88 | -4.5424 | 4.7171 |
| 38206 | B12Li4O20 | 36 | -6.8926 | 6.3945 |
| 165579 | Cl4Li4O16 | 24 | -3.5159 | 5.7902 |
| 245516 | Li8O28Si12 | 48 | -6.3294 | 5.7291 |
| 32595 | Al12Li12O48Si12 | 84 | -6.3958 | 5.5552 |
| 28288 | Al3Li3O6 | 12 | -6.0016 | 7.0686 |
| 159536 | Li4O24Sc4Si8 | 40 | -6.7698 | 5.0984 |
| 69763 | Ge12Li6O72P18 | 108 | -5.6955 | 3.9812 |
| 75161 | Ca8Li4O26Si8 | 46 | -6.3843 | 5.1729 |
| 67238 | Al18Ge18Li18O72 | 126 | -5.7833 | 3.9818 |
| 163914 | La24Li56O96Sn16 | 192 | -5.6386 | 4.0248 |
| 422922 | B48Li16O80 | 144 | -6.9071 | 6.0688 |
| 97909 | Al4Li4O16Si4 | 28 | -6.3778 | 5.7643 |
| 65175 | Ge2Li16O12 | 30 | -4.585 | 4.6818 |
| 421479 | Cl4Li24O20P4 | 52 | -4.8075 | 6.1974 |
| 90849 | B8Li8O48Si16 | 80 | -6.6938 | 6.7022 |
| 67239 | Ga18Ge18Li18O72 | 126 | -5.2736 | 3.6717 |
| 28388 | Ga3Li3O6 | 12 | -5.0705 | 4.8568 |
| 73241 | Li4Mg4O22Si8 | 38 | -6.2544 | 5.9713 |
| 92842 | B4Li4O12Sr4 | 24 | -6.1941 | 5.4545 |
| 62333 | In12Li18O72P18 | 120 | -5.4881 | 4.4518 |
| 174007 | Al2Li2O20Si8 | 32 | -6.6833 | 5.8841 |
| 9987 | B4Ga2Li6O12 | 24 | -5.8993 | 5.3611 |
| 68463 | Ge10Li8O24 | 42 | -5.2865 | 3.9598 |
| 30909 | Ba8Li12O84P28 | 132 | -5.8349 | 6.0635 |
| 65177 | Ge4Li16O16 | 36 | -4.8522 | 5.0752 |
| 41433 | Ba8Li16O56P16 | 96 | -5.7914 | 6.3836 |
| 68247 | Ge28Li8O60 | 96 | -5.3896 | 3.8247 |
| 87990 | Al6Cl2Ge6Li8O24 | 46 | -5.6018 | 4.9526 |

Table S2. Materials ID of extracted DFT dataset from 1000 Li-containing oxides from Materials Project (Jain et al., Comp. Mater. Sci. 2011;50:2295-2310).

| mp-10499 | mp-27111 | mp-558294 | mp-672993 | mp-753327 | mp-757431 | mp-759329 | mp-766374 | mp-769374 | mp-774797 |
| --- | --- | --- | --- | --- | --- | --- | --- | --- | --- |
| mp-10517 | mp-27122 | mp-558310 | mp-673023 | mp-753349 | mp-757434 | mp-759360 | mp-766433 | mp-769380 | mp-774832 |
| mp-10814 | mp-27275 | mp-558312 | mp-673024 | mp-753363 | mp-757464 | mp-759377 | mp-766449 | mp-769384 | mp-774838 |
| mp-11189 | mp-27417 | mp-558382 | mp-673029 | mp-7535 | mp-757466 | mp-759381 | mp-766457 | mp-769411 | mp-774859 |
| mp-11206 | mp-27483 | mp-558455 | mp-673059 | mp-753511 | mp-757479 | mp-759382 | mp-766543 | mp-770645 | mp-774908 |
| mp-11737 | mp-27767 | mp-558483 | mp-673073 | mp-753544 | mp-757490 | mp-759384 | mp-766548 | mp-770647 | mp-774910 |
| mp-13182 | mp-27811 | mp-558628 | mp-673090 | mp-753580 | mp-757505 | mp-759390 | mp-766823 | mp-770648 | mp-775424 |
| mp-13725 | mp-27968 | mp-558713 | mp-673098 | mp-753639 | mp-757527 | mp-759396 | mp-766828 | mp-770649 | mp-775429 |
| mp-13843 | mp-28016 | mp-558808 | mp-673101 | mp-753649 | mp-757532 | mp-759406 | mp-766834 | mp-770654 | mp-775484 |
| mp-14030 | mp-28146 | mp-558890 | mp-673119 | mp-753654 | mp-757541 | mp-759455 | mp-766983 | mp-770700 | mp-775486 |
| mp-14232 | mp-28253 | mp-558941 | mp-673126 | mp-753719 | mp-757542 | mp-759485 | mp-767086 | mp-770731 | mp-775796 |
| mp-14363 | mp-28450 | mp-559052 | mp-673134 | mp-753795 | mp-757547 | mp-759498 | mp-767091 | mp-770743 | mp-775916 |
| mp-14364 | mp-28510 | mp-559070 | mp-673144 | mp-753864 | mp-757560 | mp-759534 | mp-767146 | mp-770756 | mp-775971 |
| mp-14399 | mp-28548 | mp-559106 | mp-673156 | mp-754060 | mp-757569 | mp-759550 | mp-767194 | mp-770759 | mp-776021 |
| mp-14495 | mp-28549 | mp-559142 | mp-674176 | mp-754206 | mp-757578 | mp-759560 | mp-767214 | mp-770799 | mp-776108 |
| mp-14704 | mp-28592 | mp-559300 | mp-674361 | mp-754249 | mp-757590 | mp-759568 | mp-767295 | mp-770805 | mp-776215 |
| mp-14854 | mp-28593 | mp-559441 | mp-6745 | mp-754344 | mp-757597 | mp-759596 | mp-767341 | mp-770808 | mp-776371 |
| mp-14871 | mp-28623 | mp-559452 | mp-675069 | mp-754345 | mp-757616 | mp-759607 | mp-767384 | mp-770833 | mp-776484 |
| mp-15349 | mp-2878 | mp-559533 | mp-675334 | mp-754445 | mp-757658 | mp-759653 | mp-767392 | mp-770884 | mp-776502 |
| mp-15543 | mp-28891 | mp-559554 | mp-675379 | mp-754519 | mp-757663 | mp-759667 | mp-767393 | mp-770932 | mp-776510 |
| mp-15960 | mp-29077 | mp-559612 | mp-675606 | mp-754586 | mp-757671 | mp-759680 | mp-767424 | mp-770997 | mp-776589 |
| mp-16691 | mp-29195 | mp-559627 | mp-675692 | mp-754723 | mp-757673 | mp-759696 | mp-767490 | mp-771028 | mp-776625 |
| mp-16792 | mp-2931 | mp-559689 | mp-675734 | mp-754751 | mp-757690 | mp-759713 | mp-767497 | mp-771334 | mp-777071 |
| mp-16804 | mp-531409 | mp-559715 | mp-676365 | mp-754801 | mp-757734 | mp-759715 | mp-767498 | mp-771341 | mp-777167 |
| mp-16828 | mp-531523 | mp-559767 | mp-6765 | mp-754822 | mp-757758 | mp-759734 | mp-767511 | mp-771707 | mp-777356 |
| mp-16947 | mp-531722 | mp-559848 | mp-676694 | mp-754902 | mp-757765 | mp-759784 | mp-767604 | mp-771710 | mp-777751 |
| mp-17208 | mp-531820 | mp-559904 | mp-676723 | mp-754937 | mp-757777 | mp-759849 | mp-767633 | mp-771804 | mp-777851 |
| mp-17672 | mp-531914 | mp-559908 | mp-677136 | mp-755000 | mp-757781 | mp-759868 | mp-767674 | mp-771832 | mp-777931 |
| mp-17718 | mp-532676 | mp-559934 | mp-677212 | mp-755013 | mp-757788 | mp-759871 | mp-768114 | mp-771979 | mp-778009 |
| mp-17774 | mp-540945 | mp-559971 | mp-677305 | mp-755144 | mp-757793 | mp-759915 | mp-768142 | mp-772030 | mp-778109 |
| mp-17854 | mp-541661 | mp-559987 | mp-677510 | mp-755225 | mp-757806 | mp-759918 | mp-768150 | mp-772049 | mp-778111 |
| mp-18002 | mp-5418 | mp-560036 | mp-6782 | mp-755231 | mp-757816 | mp-759926 | mp-768153 | mp-772055 | mp-778115 |
| mp-18037 | mp-542874 | mp-560072 | mp-6783 | mp-755253 | mp-757817 | mp-759933 | mp-768159 | mp-772073 | mp-778150 |
| mp-18048 | mp-545343 | mp-560189 | mp-6787 | mp-755256 | mp-757838 | mp-760017 | mp-768160 | mp-772085 | mp-778397 |
| mp-18147 | mp-545346 | mp-560209 | mp-6800 | mp-755266 | mp-757845 | mp-760093 | mp-768161 | mp-772108 | mp-778460 |
| mp-18220 | mp-546920 | mp-560247 | mp-681439 | mp-755289 | mp-757874 | mp-760348 | mp-768164 | mp-772109 | mp-778604 |
| mp-18422 | mp-5488 | mp-560463 | mp-683616 | mp-755298 | mp-757883 | mp-760377 | mp-768169 | mp-772132 | mp-778877 |
| mp-18495 | mp-549207 | mp-560614 | mp-684028 | mp-755346 | mp-757884 | mp-760378 | mp-768185 | mp-772147 | mp-778878 |
| mp-18640 | mp-549308 | mp-560653 | mp-684042 | mp-755392 | mp-757885 | mp-760408 | mp-768190 | mp-772150 | mp-779010 |
| mp-1960 | mp-549389 | mp-560667 | mp-684052 | mp-755487 | mp-757889 | mp-760462 | mp-768220 | mp-772151 | mp-779116 |
| mp-22170 | mp-549565 | mp-560739 | mp-684059 | mp-755505 | mp-757890 | mp-760539 | mp-768256 | mp-772155 | mp-779455 |
| mp-22450 | mp-550320 | mp-560894 | mp-684066 | mp-755559 | mp-757899 | mp-760594 | mp-768284 | mp-772157 | mp-779482 |
| mp-22538 | mp-550474 | mp-561011 | mp-684082 | mp-755613 | mp-757901 | mp-760629 | mp-768292 | mp-772176 | mp-779525 |
| mp-22694 | mp-550498 | mp-561237 | mp-684090 | mp-755615 | mp-757905 | mp-760637 | mp-768437 | mp-772185 | mp-779602 |
| mp-22955 | mp-550798 | mp-561254 | mp-684093 | mp-755691 | mp-757914 | mp-760650 | mp-768452 | mp-772212 | mp-779809 |
| mp-22994 | mp-551740 | mp-561294 | mp-684104 | mp-755703 | mp-757945 | mp-760655 | mp-768609 | mp-772214 | mp-780235 |
| mp-23582 | mp-552588 | mp-561336 | mp-684108 | mp-755707 | mp-757946 | mp-760690 | mp-768636 | mp-772215 | mp-780333 |
| mp-23648 | mp-552663 | mp-561474 | mp-684109 | mp-755745 | mp-757947 | mp-760701 | mp-768665 | mp-772216 | mp-780576 |
| mp-25400 | mp-553926 | mp-562137 | mp-684119 | mp-755757 | mp-757964 | mp-760832 | mp-768678 | mp-772220 | mp-780724 |
| mp-25401 | mp-553950 | mp-5670 | mp-684427 | mp-755783 | mp-757967 | mp-7610 | mp-768731 | mp-772416 | mp-781626 |
| mp-25417 | mp-554003 | mp-5769 | mp-684446 | mp-755835 | mp-757974 | mp-7611 | mp-768736 | mp-772521 | mp-781788 |
| mp-25418 | mp-554159 | mp-5832 | mp-684477 | mp-755894 | mp-757996 | mp-761135 | mp-768738 | mp-772523 | mp-781803 |
| mp-25420 | mp-554164 | mp-584134 | mp-684489 | mp-755959 | mp-757997 | mp-761143 | mp-768740 | mp-772524 | mp-782005 |
| mp-25462 | mp-554344 | mp-585241 | mp-684506 | mp-756035 | mp-758002 | mp-761144 | mp-768747 | mp-772535 | mp-782065 |
| mp-25802 | mp-554560 | mp-585305 | mp-684508 | mp-756045 | mp-758026 | mp-761687 | mp-768755 | mp-772545 | mp-7941 |
| mp-25839 | mp-554577 | mp-5854 | mp-684509 | mp-756117 | mp-758031 | mp-761694 | mp-768756 | mp-772619 | mp-7971 |
| mp-25881 | mp-554752 | mp-585408 | mp-684534 | mp-756170 | mp-758035 | mp-761773 | mp-768764 | mp-772710 | mp-7998 |
| mp-25907 | mp-554917 | mp-5990 | mp-684539 | mp-756294 | mp-758037 | mp-761825 | mp-768765 | mp-772721 | mp-8001 |
| mp-25929 | mp-555082 | mp-6015 | mp-684817 | mp-756299 | mp-758056 | mp-761841 | mp-768771 | mp-772750 | mp-8070 |
| mp-25935 | mp-555089 | mp-605853 | mp-685194 | mp-756306 | mp-758063 | mp-761849 | mp-768776 | mp-772783 | mp-8179 |
| mp-25959 | mp-555140 | mp-6075 | mp-685320 | mp-756344 | mp-758086 | mp-761857 | mp-768785 | mp-772826 | mp-8180 |
| mp-26113 | mp-555189 | mp-6097 | mp-685413 | mp-756406 | mp-758089 | mp-761862 | mp-768788 | mp-772899 | mp-8184 |
| mp-26129 | mp-555256 | mp-6099 | mp-685675 | mp-756432 | mp-758115 | mp-761891 | mp-768789 | mp-772920 | mp-8294 |
| mp-26157 | mp-555320 | mp-6113 | mp-685797 | mp-756515 | mp-758140 | mp-761922 | mp-768794 | mp-772925 | mp-8313 |
| mp-26173 | mp-555420 | mp-6126 | mp-686041 | mp-756557 | mp-758145 | mp-761952 | mp-768795 | mp-772972 | mp-841 |
| mp-26209 | mp-555489 | mp-613442 | mp-686057 | mp-756560 | mp-758151 | mp-762044 | mp-768797 | mp-772979 | mp-8449 |
| mp-26245 | mp-555743 | mp-6146 | mp-686097 | mp-756628 | mp-758218 | mp-762240 | mp-768798 | mp-772993 | mp-8450 |
| mp-26271 | mp-555899 | mp-6179 | mp-686160 | mp-756639 | mp-758224 | mp-762248 | mp-7688 | mp-772997 | mp-8491 |
| mp-26281 | mp-555958 | mp-6211 | mp-686230 | mp-756641 | mp-758240 | mp-762322 | mp-768809 | mp-773024 | mp-849265 |
| mp-26285 | mp-555965 | mp-6224 | mp-686484 | mp-756645 | mp-758245 | mp-762329 | mp-768812 | mp-773039 | mp-849732 |
| mp-26299 | mp-556066 | mp-6240 | mp-686745 | mp-756660 | mp-758270 | mp-762336 | mp-768821 | mp-773049 | mp-849779 |
| mp-26301 | mp-556137 | mp-6241 | mp-6944 | mp-756684 | mp-758287 | mp-762351 | mp-768875 | mp-773052 | mp-850185 |
| mp-26341 | mp-556165 | mp-6259 | mp-694958 | mp-756718 | mp-758310 | mp-762355 | mp-768884 | mp-773068 | mp-850190 |
| mp-26389 | mp-556215 | mp-630234 | mp-695000 | mp-756755 | mp-758376 | mp-762500 | mp-768890 | mp-773074 | mp-850532 |
| mp-26407 | mp-556531 | mp-6318 | mp-695259 | mp-756768 | mp-758447 | mp-7638 | mp-768921 | mp-773078 | mp-851102 |
| mp-26409 | mp-556649 | mp-6326 | mp-695365 | mp-756781 | mp-758492 | mp-765093 | mp-768947 | mp-773081 | mp-851118 |
| mp-26541 | mp-556777 | mp-6327 | mp-695366 | mp-756793 | mp-758533 | mp-765102 | mp-768960 | mp-773084 | mp-851120 |
| mp-26549 | mp-556783 | mp-632815 | mp-695434 | mp-756818 | mp-758651 | mp-765150 | mp-768966 | mp-773089 | mp-863362 |
| mp-26586 | mp-556786 | mp-6332 | mp-695505 | mp-756853 | mp-758731 | mp-765193 | mp-768968 | mp-773101 | mp-863365 |
| mp-26591 | mp-556861 | mp-6340 | mp-696125 | mp-756854 | mp-758770 | mp-765222 | mp-768970 | mp-773129 | mp-863377 |
| mp-26629 | mp-556886 | mp-636952 | mp-696130 | mp-756908 | mp-758780 | mp-765227 | mp-768971 | mp-773843 | mp-863382 |
| mp-26649 | mp-556902 | mp-641919 | mp-696136 | mp-756912 | mp-758855 | mp-765243 | mp-768986 | mp-774064 | mp-863403 |
| mp-26650 | mp-557104 | mp-6425 | mp-696822 | mp-756935 | mp-758894 | mp-765277 | mp-768987 | mp-7744 | mp-863408 |
| mp-26652 | mp-557112 | mp-6442 | mp-706276 | mp-756951 | mp-758944 | mp-765286 | mp-768992 | mp-774449 | mp-863410 |
| mp-26675 | mp-557328 | mp-644527 | mp-720245 | mp-757038 | mp-759028 | mp-765289 | mp-769001 | mp-774474 | mp-863427 |
| mp-26701 | mp-557375 | mp-645317 | mp-7205 | mp-757042 | mp-759029 | mp-765351 | mp-769002 | mp-774479 | mp-865750 |
| mp-26710 | mp-557395 | mp-6456 | mp-721238 | mp-757047 | mp-759068 | mp-765573 | mp-769004 | mp-774482 | mp-8673 |
| mp-26721 | mp-557456 | mp-6499 | mp-721249 | mp-757048 | mp-759071 | mp-765985 | mp-769006 | mp-774528 | mp-867533 |
| mp-26725 | mp-557467 | mp-650121 | mp-721255 | mp-757053 | mp-759099 | mp-766028 | mp-769008 | mp-774552 | mp-867613 |
| mp-26745 | mp-557473 | mp-652479 | mp-735824 | mp-757082 | mp-759117 | mp-766047 | mp-769010 | mp-774554 | mp-867649 |
| mp-26767 | mp-557738 | mp-6553 | mp-735910 | mp-757089 | mp-759122 | mp-766084 | mp-769026 | mp-774579 | mp-867681 |
| mp-26835 | mp-557756 | mp-6565 | mp-7471 | mp-757106 | mp-759128 | mp-766103 | mp-769040 | mp-774609 | mp-867727 |
| mp-26855 | mp-557857 | mp-6597 | mp-752501 | mp-757142 | mp-759141 | mp-766170 | mp-769069 | mp-774623 | mp-8702 |
| mp-26856 | mp-557861 | mp-6648 | mp-752546 | mp-757158 | mp-759187 | mp-766176 | mp-769073 | mp-774658 | mp-8796 |
| mp-26871 | mp-558054 | mp-6663 | mp-752636 | mp-757219 | mp-759227 | mp-766179 | mp-769074 | mp-774697 | mp-8870 |
| mp-26884 | mp-558082 | mp-6668 | mp-752879 | mp-757282 | mp-759251 | mp-766196 | mp-769078 | mp-774702 | mp-8874 |
| mp-26949 | mp-558083 | mp-667309 | mp-752922 | mp-757305 | mp-759254 | mp-766202 | mp-769129 | mp-774714 | mp-9018 |
| mp-27017 | mp-558102 | mp-672958 | mp-752926 | mp-757325 | mp-759263 | mp-766208 | mp-769287 | mp-774715 | mp-9172 |
| mp-27069 | mp-558105 | mp-672976 | mp-753162 | mp-757327 | mp-759289 | mp-766251 | mp-769316 | mp-774742 | mp-9197 |
| mp-27093 | mp-558256 | mp-672979 | mp-753319 | mp-757383 | mp-759322 | mp-766350 | mp-769319 | mp-774753 | mp-9625 |
